# Increased serum concentrations of IL-1 beta, IL-21 and Th17 cells in overweight patients with rheumatoid arthritis

**DOI:** 10.1186/s13075-017-1308-y

**Published:** 2017-05-31

**Authors:** Hirofumi Shoda, Yasuo Nagafuchi, Yumi Tsuchida, Keiichi Sakurai, Shuji Sumitomo, Keishi Fujio, Kazuhiko Yamamoto

**Affiliations:** Department of Allergy and Rheumatology, Graduate School of Medicine, the University of Tokyo, 7-3-1 Hongo, Bunkyo-ku, Tokyo, Japan

**Keywords:** Rheumatoid arthritis, Body mass index, Th17, IL-1β, IL-21

## Abstract

**Backgrounds:**

Obesity is associated with worse disease activity and drug responses in patients with rheumatoid arthritis (RA). However, the immunological mechanisms responsible for the relationship between RA and obesity have not yet been clarified in detail. This study aimed to elucidate the immunological mechanisms contributing to the pathogenesis of RA in overweight patients.

**Methods:**

The frequencies of CD4^+^ T cell, B cell and monocyte subsets were analyzed in RA (n = 81) and healthy donors (n = 99) by flow cytometry, and were compared between three groups (body mass index (BMI) <20,  ≥20 to 25, >25). Serum cytokines were measured using multiplex ELISA. Gene expression was analyzed by quantitative PCR. Clinical information was extracted from medical records.

**Results:**

The frequencies of T helper (Th)17 (CD4^+^CD45RA-CXCR5-CXCR3-CCR6^+^) cells and plasmablasts (PB) were significantly increased in patients with RA with BMI >25. Significant correlation was observed between BMI and Th17 cells in patients with RA. No significant differences in cell frequencies between the three BMI groups were observed in the healthy donors. Serum interleukin (IL)-1β and IL-21 significantly correlated with BMI in RA patients. Gene expression patterns in Th17 cells from overweight patients with RA showed the characteristics of pathogenic Th17 cells.

**Conclusions:**

Quantitative and qualitative changes in Th17 cells were characteristic in overweight patients with RA.

**Electronic supplementary material:**

The online version of this article (doi:10.1186/s13075-017-1308-y) contains supplementary material, which is available to authorized users.

## Background

Rheumatoid arthritis (RA) is caused by genetic and environmental factors. In addition, some environmental factors, such as smoking, markedly affect the prognosis of patients with RA. Obesity has also been proposed as one of the environmental risk factors for RA. Obesity modifies the disease course of RA, and also results in poor responses to biological therapy [1, 2]. Cohort studies have demonstrated worse disease activity in obese patients with early RA [3]. Patients with RA who have a high body mass index (BMI) were recently reported to have worse long-term outcomes in disease activity, function, and comorbidities [4]. However, other studies have provided paradoxical findings in which the radiographic evidence of progression of bone damage was slower in obese patients with RA [5, 6]. Therefore, the influence of obesity on RA remains controversial.

Several immune cells orchestrate and play important roles in the pathogenesis of RA. We previously performed immunological cell typing in patients with RA and healthy donors (HDs) [7]. Using flow cytometry, the frequencies of peripheral T cell and B cell subsets and monocytes were measured and analyzed based on associations with several clinical parameters, including disease activity. In the previous study, the frequencies of the peripheral immune cells were compared, and some differences were identified, such as an increase of CD14^+^CD16^+^ monocytes and decreases of T helper (Th)17.1 cells, memory B cells in patients with RA [7]. In the present study, we analyzed this database in view of their relationship with BMI in order to identify the immunological features in overweight patients with RA.

## Methods

### Patients with RA and healthy donors

We recruited 81 patients with RA between April 2013 and March 2015, who fulfilled the 2010 American College of Rheumatology/European League Against Rheumatism classification criteria [8], and 99 HDs. The comparison of age, sex distribution, and BMI between patients with RA and HDs are indicated in Additional file 1: Table S1. All the donors were without active infection or malignancy. The following clinical data were collected: age, sex, disease duration, BMI, methotrexate (MTX) usage, biological disease-modifying anti-rheumatic drug (bDMARD) usage, rheumatoid factor (RF) titer, anti-cyclic citrullinated peptide (CCP) antibody titer, Disease Activity Score 28 joint-erythrocyte sedimentation rate (DAS28esr), the clinical disease activity index (CDAI) and Health Assessment Questionnaire (HAQ) Disability Index. RF titers were measured using latex coagulating nephelometry (cutoff value of 15 IU/ml). Anti-CCP antibody titers were measured using a chemiluminescence enzyme immunoassay (cutoff value of 4.5 U/ml, Medical and Biological Laboratories, Japan). All donors provided written informed consent, and the use of human peripheral blood samples was approved by the Ethical Committee of the University of Tokyo Hospital (number 10154 and G3582). The methods of the present study were performed in accordance with the approved guidelines.

### Fluorescence-activated cell sorting (FACS) and immunophenotyping

All collected blood samples were freshly analyzed by flow cytometry. Human peripheral blood mononuclear cells were isolated by Ficoll-Paque Plus density gradient centrifugation (GE Healthcare). The following antibodies were used: Human Fc Receptor Binding Inhibitor Purified (eBioscience), CD3-PE-Cy7 (UCHT1, BioLegend), CD3-PerCP-Cy5.5 (UCHT1, BioLegend), CD4-PerCP-Cy5.5 (OKT4, BioLegend), CD4-V500 (RPA-T4, BD Biosciences), CD14-FITC (M5E2, BioLegend), CD16-PerCP-Cy5.5 (3G8, BioLegend), CD19-APC-Cy7 (HIB19, BioLegend), CD19-V500 (HIB19, BD Biosciences), CD25-Brilliant Violet 421 (BC96, BioLegend), CD25-PE-Cy7 (BC96, eBioscience), CD27-FITC (O323, eBioscience), CD38-PE-Cy7 (HIT2, BioLegend), CD45RA-APC-Cy7 (HI100, BioLegend), CXCR3-Brilliant Violet 421 (1C6, BD Biosciences), CXCR5-Alexa Fluor 488 (RF8B2, BD Biosciences), and CCR6-PE (11A9, BD Biosciences). Flow cytometric analysis and cell sorting were performed on an 8-color MoFlo XDP (Beckman Coulter).

Subset definitions were described in a previous study. We classified CD4^+^ T cell and B cell subsets and monocytes based on the Human Immunology Project classification [9] and also added modifications for subsets already reported to be important in RA [7]. According to the Human Immunology Project classification [9], Th17 cells were defined as CD3^+^ CD4^+^ CD25- CD45RA- CXCR5- CXCR3- CCR6^+^ cell population. Th17.1 cells were defined as CD3^+^ CD4^+^ CD25- CD45RA- CXCR5- CXCR3^+^ CCR6^+^ cell population, which were reported as one of the Th populations which exhibited both Th1 and Th17 features [10, 11]. Detailed gating strategies were indicated in the previous article [8].

### Multiplex cytokine analysis

Serum concentrations of interleukin (IL)-1β, IL-6, IL-10, IL-17A, IL-21, interferon(IFN)-γ, granulocyte macrophage-colony stimulating factor (GM-CSF) and tumor necrosis factor (TNF)-α were measured using the Milliplex MAP kit (the Human Soluble Cytokine Receptor Magnetic Bead Panel and Human High Sensitivity T Cell Magnetic Panel, Merck Millipore) and BioPlex 3D system (Bio-Rad), according to the manufacturer’s instructions. In order to reduce false amplification by heterophilic antibodies, HeteroBlock (Omega Biologicals) was added to all serum samples in order to achieve a final concentration of 150 μg/ml [8]. Serum samples for cytokine measurement were obtained from 19 patients with RA.

### Quantitative PCR

RNA was extracted from FACS-sorted Th17 (CD3^+^ CD4^+^ CD25- CD45RA- CXCR5- CXCR3- CCR6^+^) cells using the RNeasy Micro Kit (Qiagen). RNA was reverse-transcribed to cDNA with random primers (Invitrogen) and Superscript III according to the manufacturer’s protocol (Invitrogen). Quantitative real-time PCR analysis was performed using SYBR Green Master Mix (Qiagen) and the CFX Connect Real-Time PCR (Bio-Rad). The results of real-time PCR are shown in terms of relative expression to glyceraldehyde-3-phosphate dehydrogenase (GAPDH). Analyzed genes were selected on the basis of a previous study [12]. The primers used in real-time PCR are listed in Additional file 1: Table S2.

### Statistical analysis

Differences between groups were tested with one-way analysis of variance (ANOVA) and the unpaired *t* test with post-hoc Bonferroni correction. Relationships between cell frequencies and BMI were evaluated by Pearson’s correlation coefficient. Relationships between cytokines and BMI were evaluated by Spearman’s correlation coefficient. Multiple variable regression was performed to predict the frequencies of Th17 cells based on the indicated variables. *P* values <0.05 were considered to be significant.

## Results

### Immunological cell typing and BMI in patients with RA and healthy donors

**Fig. 1:**
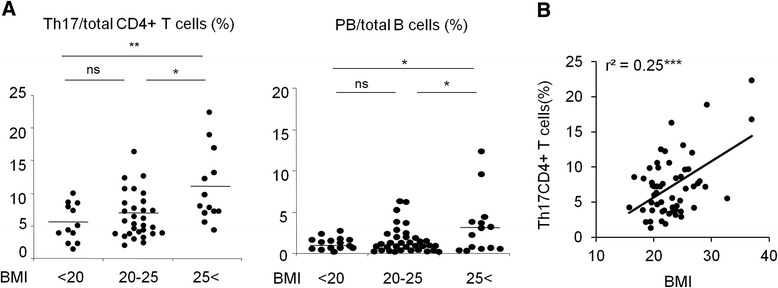
Frequencies of peripheral immune cells according to body mass index (*BMI*). **a** Comparison of the frequencies of T helper 17 (*Th17*) cells and plasmablasts (*PB*) between the three different BMI groups among patients with rheumatoid arthritis (RA). **b** The relationship between BMI and the frequencies of Th17 cells in patients with RA. Relationships were evaluated by Pearson’s correlation coefficient: *p < 0.05, **p < 0.01, ***p < 0.001, *ns* not significant

Subjects were categorized into three groups according to BMI <20,  ≥20 to 25, >25), as previously defined [1–4]. A summary of patient profiles is listed in Table 1. No significant differences were observed in clinical background, including age, disease duration, anti-CCP antibody positivity, and therapies among the three BMI groups, except for the serum titers of RF (Table 1). Disease activity scores and HAQ scores were not significantly different between the three BMI groups. The frequencies of the CD4^+^ T cell and B cell subsets and monocytes were compared between the three BMI groups among healthy donors and patients with RA (Table 2). The frequencies of Th17 cells (CD3^+^CD4^+^CD45RA-CD25-CXCR5-CXCR3-CCR6^+^) and plasmablasts (PB) were significantly different in the three BMI groups among patients with RA, and were significantly increased in the RA group with BMI >25 (Table 2 and Fig. 1a). In contrast, no significant difference was observed in immune cell frequencies in the three BMI groups among healthy donors (Table 3 and Additional file 2: Figure S1). We then investigated the relationship between BMI and immune cell frequencies. The frequency of Th17 cells positively correlated with BMI in patients with RA (*r* ^2^ = 0.25, *p* < 0.0001) (Fig. 1b). No correlations were found between PB and BMI in patients with RA (*r* ^2^ = 0.013 (PB)). The frequencies of Th17 cells were compared between patients with RA and HDs in each BMI category, and there was no significant difference (BMI <20: RA 5.40 ± 2.89, HDs 7.05 ± 2.83, *p* = 0.084, 20 < BMI <25: RA 6.44 ± 3.47, HDs 8.51 ± 2.75, *p* = 0.061, BMI >25: RA 10.68 ± 5.36, HDs 8.41 ± 1.92, *p* = 0.26). Moreover, multivariate analysis demonstrated that BMI was an independent predictor of the frequencies of Th17 cells in patients with RA (Table 4). Taken together, these results revealed immunological characteristics of overweight patients with RA, particularly a significant increase in Th17 cells.

**Table 1:** Summary of the clinical information on the patients with rheumatoid arthritis

	BMI <20	20 < BMI <25	BMI >25	*P* value
Clinical data				
Number	16	46	19	
Female (%)	81.3%	73.9%	84.2%	
Age (years)	56.9 ± 17.3	64.2 ± 11.0	59.3 ± 11.5	ns
Disease duration (months)	66.0 ± 110.1	72.4 ± 140.5	72.4 ± 171.1	ns
DAS28esr	4.67 ± 1.79	4.53 ± 1.33	4.93 ± 1.62	ns
CDAI	19.5 ± 12.9	17.4 ± 10.7	21.6 ± 16.1	ns
HAQ	0.87 ± 0.77	1.22 ± 0.92	1.39 ± 0.99	ns
Anti-CCP antibody (%)	87.5%	84.7%	89.4%	ns
RF (U/ml)	145.3 ± 295.8	164.9 ± 311.3	445.1 ± 649.7	0.035*
MTX (mg/week)	9.5 ± 2.5	9.0 ± 2.9	8.0 ± 3.11	ns
PSL dose (mg/day)	3.5 ± 4.1	3.6 ± 6.7	3.68 ± 3.89	ns
bDMARDs (%)	12.5%	13.0%	15.7%	ns

Data are shown as mean +/− SD, unless stated otherwise. Differences were analyzed by one-way analysis of variance. *Abbreviations*: *BMI* body mass index, *DAS* disease activity score, *CDAI* Clinical Disease Activity Index, *HAQ* Health Assessment Questionnaire, *CCP* cyclic-citrullinated peptide, *RF* rheumatoid factor, *MTX* methotrexate, *PSL* prednisolone, *bDMARD* biological disease-modifying anti-rheumatic drug, *ns* not significant. *P < 0.05

**Table 2:** Summary of peripheral immune cell frequencies in patients with rheumatoid arthritis

	BMI <20	20 < BMI <25	BMI >25	*P* value
CD4+ T cells (%)				
Total	44.2 ± 8.71	42.6 ± 13.1	40.3 ± 11.93	0.51
Naive T cells	32.1 ± 25.6	29.8 ± 23.7	35.7 ± 16.1	0.32
Memory T cells	26.7 ± 14.3	29.9 ± 13.2	36.7 ± 12.4	0.18
CD25 + Treg cells	6.26 ± 10.9	9.79 ± 16.4	6.19 ± 11.8	0.69
Follicular helper T cells	9.73 ± 6.05	9.95 ± 5.40	11.88 ± 6.17	0.69
Th1 cells	5.48 ± 3.18	5.50 ± 3.09	5.76 ± 2.65	0.13
Th2 cells	9.36 ± 6.81	14.52 ± 9.88	15.79 ± 5.77	0.53
Th17 cells	5.40 ± 2.89	6.44 ± 3.47	10.68 ± 5.36	0.0029**
Th17.1 cells	4.30 ± 3.38	3.71 ± 2.31	5.61 ± 4.30	0.96
B cells (%)				
Total	6.36 ± 5.75	7.70 ± 6.03	5.11 ± 3.74	0.29
Naive B cells	65.3 ± 15.0	66.3 ± 21.2	63.1 ± 17.7	0.86
Switch- memory B cells	5.36 ± 3.63	4.53 ± 3.46	3.79 ± 21.8	0.064
Switch + memory B cells	16.9 ± 8.21	16.8 ± 11.7	19.9 ± 13.6	0.50
Double negative B cells	12.5 ± 8.55	12.5 ± 11.3	12.0 ± 9.33	0.99
Plasmablast	1.59 ± 1.88	1.79 ± 2.90	3.35 ± 3.51	0.020*
Monocytes (%)				
CD14^+^ CD16-	82.3 ± 17.5	85.3 ± 10.5	86.3 ± 8.58	0.66
CD14^+^CD16^+^	4.77 ± 3.71	4.57 ± 3.16	3.49 ± 2.11	0.48
CD14^mid^CD16^+^	11.7 ± 13.9	8.85 ± 6.99	9.26 ± 6.64	0.59

The percentages of total CD4^+^ T cells and B cells are shown as a ratio to the total number of lymphocytes. The percentages of cell subsets are shown as a ratio to total CD4^+^ T cells, B cells, and monocytes. The definitions of cell subsets were based on the Human Immunology Project classification [9]. Data are shown as mean +/− SD. *BMI* body mass index, *Treg* T regulatory cells, *Th* T helper. Differences were analyzed by one-way analysis of variance: *p < 0.05, **p < 0.01

**Table 3:** Summary of peripheral immune cell frequencies in healthy donors

	BMI <20 (n = 40)	20 < BMI <25 (n = 51)	BMI >25 (n = 8)	*P* value
CD4^+^ T cells (%)				
Total	40.1 ± 9.55	40.0 ± 9.17	35.7 ± 5.32	0.46
Naive T cells	57.9 ± 12.4	51.6 ± 10.3	48.3 ± 15.1	0.056
Memory T cells	30.6 ± 10.0	34.0 ± 7.97	36.5 ± 9.77	0.077
CD25^+^Treg cells	2.83 ± 1.16	3.51 ± 1.41	3.75 ± 1.92	0.069
Follicular helper T cells	9.43 ± 3.63	11.9 ± 3.91	12.7 ± 6.35	0.083
Th1 cells	7.25 ± 3.03	7.79 ± 2.86	8.85 ± 3.37	0.34
Th2 cells	8.12 ± 3.61	8.80 ± 3.72	9.47 ± 4.72	0.54
Th17 cells	7.05 ± 2.83	8.51 ± 2.75	8.41 ± 1.92	0.066
Th17.1 cells	7.94 ± 4.72	8.87 ± 4.01	9.79 ± 6.88	0.46
B cells (%)				
Total	4.73 ± 2.91	5.15 ± 2.69	6.15 ± 2.14	0.39
Naive B cells	54.9 ± 14.6	53.5 ± 17.0	66.0 ± 11.4	0.12
Switch- memory B cells	10.4 ± 5.12	10.1 ± 4.11	8.01 ± 2.21	0.38
Switch^+^memory B cells	27.1 ± 11.6	27.5 ± 11.6	19.5 ± 7.69	0.18
Double negative B cells	7.64 ± 4.33	8.17 ± 6.65	6.48 ± 2.75	0.70
Plasmablast	4.11 ± 7.73	3.19 ± 3.78	2.16 ± 1.73	0.59
Monocytes (%)				
CD14^+^CD16−	90.7 ± 4.93	89.7 ± 4.75	88.2 ± 5.93	0.39
CD14^+^CD16+	2.02 ± 1.23	2.31 ± 1.26	3.05 ± 2.57	0.15
CD14^mid^CD16+	6.78 ± 3.69	7.33 ± 3.87	7.90 ± 3.39	0.67

The percentages of total CD4^+^ T cells and B cells are shown as a ratio to the total number of lymphocytes. The percentages of cell subsets are shown as a ratio to total CD4^+^ T cells, B cells, and monocytes. Data are shown as mean +/− SD. *BMI* body mass index, *Treg* T regulatory cells, *Th* T helper. Differences were analyzed by one-way analysis of variance: *p < 0.05

**Table 4:** Multiple variable regression was calculated to predict the frequencies of Th17 cells based on the indicated variables in patients with rheumatoid arthritis (n = 81)

Variables	β regression coefficient	*P* value
Sex	0.244	0.32
Age	−0.158	0.089
BMI	0.495	0.0011**
Disease duration	0.155	0.36
DAS28esr	−0.048	0.89
CDAI	0.120	0.73
HAQ	0.110	0.51
Anti-CCP antibody positivity	0.243	0.67
RF titer (U/mL)	−0.097	0.52
MTX user	0.218	0.11
PSL user	0.177	0.26
bDMARDs user	−0.132	0.38

*BMI* body mass index, *DAS28esr* Disease Activity Score 28 joints-erythrocyte sedimentation rate, *CDAI* Clinical Disease Activity Index, *HAQ* Health Assessment Questionnaire, *Anti-CCP* anti-citrullinated peptide, *RF* rheumatoid factor, *MTX* methotrexate, *PSL* prednisolone, *bDMARDS* biological disease-modifying anti-rheumatic drugs. **P < 0.01

### Serum IL-1β concentrations correlated with BMI in patients with RA

**Fig. 2:**
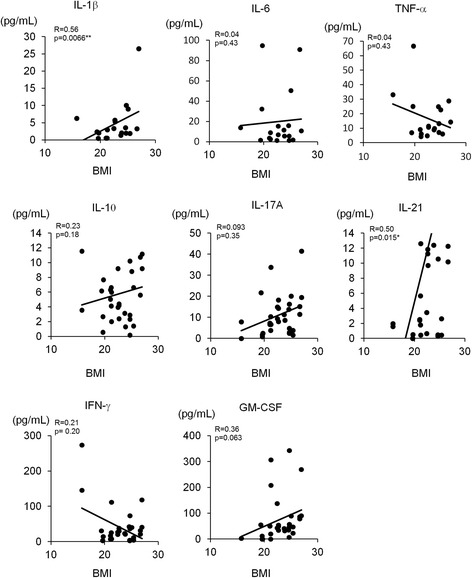
Relationship between body mass index (*BMI*) and serum cytokines in patients with rheumatoid arthritis (RA). Relationships were evaluated by Spearman’s correlation coefficient. Serum samples for cytokine measurement were obtained from 19 patients with RA. *P < 0.05. *IFN* interferon, *GM-CSF* granulocyte macrophage-colony stimulating factor

Pro-inflammatory cytokines, such as IL-1β, IL-6 and TNF-α, which are the targets of bDMARDs, play pivotal roles in RA pathogenesis. Serum pro-inflammatory cytokines were measured in patients with RA and were analyzed for correlation with BMI (Fig. 2). Serum IL-1β and IL-21 were positively correlated with BMI. In addition, serum IL-17A and GM-CSF were weakly positively correlated with BMI. In contrast, serum IFN-γ was negatively correlated with BMI. Notably, serum IL-6, IL-10 and TNF-α were not correlated with BMI (Fig. 2).

### Gene expression patterns in Th17 cells in overweight patients with RA

**Fig. 3:**
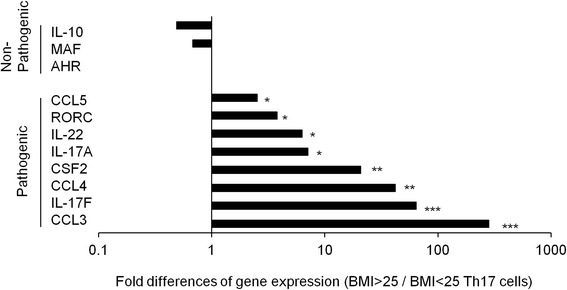
Gene expression patterns in Th17 cells from patients with rheumatoid arthritis (RA). Fold differences of gene expression patterns in Th17 cells were indicated between patients with body mass index (*BMI*) < 25 and BMI >25 among patients with RA (n = 3, respectively). *P < 0.05, **P < 0.01, ***P < 0.001

Gene expression patterns in Th17 cells were analyzed in overweight and non-overweight patients with RA (Fig. 3). The characteristic gene expression patterns in pathogenic Th17 cells, including pro-inflammatory cytokines and chemokines (CSF-2 (GM-CSF), IL-17A, IL-17 F, IL-22, CCL3, CCL4, and CCL5) [12], were significantly increased in the Th17 cells of overweight patients with RA. In contrast, the characteristic gene expression patterns in non-pathogenic Th17 cells, such as IL-10, AHR, and, MAF [12], were slightly decreased in the Th17 cells of the overweight patients with RA. These results demonstrated that not only the quantity, but also the quality of Th17 cells had pathogenically changed in the overweight patients with RA.

## Discussion

The pathological role of obesity in RA remains unclear. Some studies have reported radiographic evidence of protective effects of obesity, while others have demonstrated worse clinical disease course and drug responses to anti-TNF therapy in obese patients with RA [1–4]. Few studies have investigated the mechanisms responsible for the relationship between BMI and the RA disease course. In our study, there was no significant correlation between BMI and ongoing disease activity and the HAQ score in patients with RA. As we reported previously, disease activities were correlated with T cell activation via T cell receptor signaling [7], and we thought that these factors mainly contributed to the ongoing inflammation. RA is thought to be a heterogeneous disease and the immunological abnormalities also differ from patients to patients [13]. For example, TNF-inhibitor-resistant patients with RA sometimes respond to anti-IL-6 therapy or anti-T-cell therapy [14]. These clinical observations support the idea that the immunological abnormalities were heterogeneous in patients with RA. We demonstrated the upregulation of serum IL-1β, IL-21 and Th17 cells in overweight patients with RA. In this study, we defined Th17 cells according to the Human Immunology Project classification [9], and it was reported that IL-17-producing cells were markedly enriched in this population [15]. Notably, the significant correlation between Th17 cells and BMI was only observed in patients with RA, not in HDs. This fact suggested a possibility that the RA-specific inflammatory process would amplify the fat-oriented inflammatory process in the overweight patients with RA. Therefore, the overweight patients with RA were supposed to be a subgroup of patients with RA with the characteristic immunological phenotypes. IL-1β, IL-21 and Th17 cells play important roles in the pathogenesis of RA in several aspects. IL-1β is one of the representative pro-inflammatory cytokines in RA and blockade of IL-1 is one of the therapeutic choices [16]. IL-21 was reported as an inducer of Th17 cells in RA synovium [17]. Especially, the pathological roles of Th17 cells in RA were reported, such as secreting pro-inflammatory cytokines [17, 18]. In this way, the upregulation of Th17 cells could contribute to the pathogenesis of RA in the overweight patients. As the relationship between obesity and progression of bone damage is controversial [1–6], the relationship between BMI and radiological prognos is needs to be elucidated in several different cohorts.

Obesity has recently been thought to be a chronic inflammation process. Adipose tissue is an endocrine/paracrine organ and secretes pro-inflammatory cytokines. Adipose tissue also secretes adipokines, which have been reported to be increased in patients with RA and have been associated with joint damage [19]. For example, leptin has been identified as an inducer of Th17 cells, and hit as been shown to exacerbate collagen-induced arthritis (CIA) by enhancing Th17 cell responses [20]. Resistin-like molecule α is an adipokine that induces Th17 cells in the colitis model [21]. Visfatin strongly induces IL-6, IL-8, and matrix metalloproteinases by synovial fibroblasts [22]. Although we did not include these adipokines in this study, they could relate to the pathogenesis of the overweight patients with RA. Furthermore, free fatty acid also sensitizes dendritic cells to induce Th17 response, and could be a possible mediator in RA pathogenesis [23]. These points will be addressed in the future.

A recent report suggested that nucleotide-binding domain, leucine-rich containing family, pyrin domain-containig-3 (Nlrp3) inflammasome plays a pivotal role in obesity-induced chronic inflammation, and Nlrp3 inflammasome activates caspase-1, which induces the secretion of IL-1β [24]. These findings support the idea that being overweight is associated with chronic systemic inflammation and pro-inflammatory phenotypes in patients with RA, such as an increase in serum IL-1β, and may affect the clinical disease course and drug responses in obese patients with RA. To address this point further, body fat measurement is more appropriate than BMI, and the association between body fat mass and the immunological parameters will be elucidated. In addition, obesity plays several roles in inflammatory processes in the joints. For example, mechanical joint damage is more evident in obese people, and this kind of minor trauma is thought to induce the inflammation in the joints [6], which would cause excess cytokine secretions and Th17 differentiations in RA.

In the present study, Th17 cells were increased in the overweightp with RA. In mouse models, obesity was shown to aggravate CIA by promoting Th17 cell differentiation [25]. Moreover, gene expression patterns in Th17 cells had the characteristics of pathogenic Th17 cells in the overweight patients with RA. The importance of Th17 cells in the pathogenesis of RA has already been demonstrated [18, 26, 27]. Pathogenic Th17 cells secrete pro-inflammatory cytokines, such as IL-21, which play pivotal roles in the synovial inflammation [18]. IL-1β is crucially involved in the differentiation and pathogenicity of human Th17 cells [28]. These findings suggest that the chronic inflammation in the adipose tissue contributed to the increases in serum IL-1β and induced the differentiation of pathogenic Th17 cells in the overweight patients with RA. The obese patients with RA have exhibited worse responses to TNF-α inhibitors [1, 2]. These findings have contributed to the individualized selection of bDMARDs for patients with RA, and an anti-IL-1β, anti-IL-21 and anti-Th17 cell strategy may represent a better option for the obese patients with RA. In the case of obese psoriatic arthritis, weight reduction was associated with greater response to TNF-inhibitor [29], and weight reduction will also be a reasonable approach to recover the response to the therapies in patients with RA.

Limitations of this study were as follows: (1) the analysis of Th17 subset was based on CCR expression, but not on the production of IL-17; (2) the sample sizes were relatively small in some studies, including the gene expression study, which was only based on BMI <25/>25; (3) serum cytokines were not measured in the whole patient group; (4) the study subjects were the Japanese population; (5) the other adipokines, such as adiponectin and leptin, were not measured;and (6) age-matched healthy controls were missing. These points will be elucidated in future.

## Conclusions

In conclusion, BMI was closely associated with chronic systemic inflammation in patients with RA, and quantitative and qualitative changes in Th17 cells were observed in overweight patients with RA. We assumed that the overweight patients with RA were a subgroup of patients with RA who had the characteristic immunological phenotypes. These results provide insights into the immunological mechanisms underlying worse clinical courses and drug responses in obese patients. Individualized therapy will be addressed on the basis of these observations.

## Additional files

Additional file 1: Table S1. Comparison of age, sex, and BMI between rheumatoid arthritis (RA) patients and healthy donors (HDs). Data are indicated as mean +/- SD. Age and BMI were compared by the unpaired *t* test, and sex distribution was compared by chi-square test. **Table S2.** List of the primers (DOCX 15 kb)

Additional file 2: Figure S1. Frequencies of peripheral immune cells (Th17 cells and Plasmablast (PB)) and BMI. A comparison of the frequencies of Th17 cells and PB between the three BMI groups among healthy donors. A *p* value <0.05 was defined as a significant difference. *ns* not significant (TIF 569 kb)

## Abbreviations

bDMARD: biological disease-modifying anti-rheumatic drug
BMI: Body mass index
CCP: Cyclic citrullinated peptide
CDAI: Clinical Disease Activity Index
CIA: Collagen-induecd arthritis
DAS: Disease Activity Score
DAS28esr: Disease Activity Score 28 joint-erythrocyte sedimentation rate
ESR: Erythrocyte sedimentation rate
FACS: fluorescence-activated cell sorting
GM-CSF: Granulocyte macrophage-colony stimulating factor
HAQ: Health Assessment Questionnaire Disability Index
HD: Healthy donor
IFN: Interferon
IL: Interleukin
MTX: Methotrexate
Nlrp3: Nucleotide-binding domein, leucine-rich containing family, pyrin domain-containing-3
PB: plasmablasts
PSL: prednisolone
RA: Rheumatoid arthritis
RF: Rheumatoid factor
Th: T helper
TNF: Tumor necrosis factor
