# Supplementary material for: Increased serum concentrations of IL-1 beta, IL-21 and Th17 cells in overweight patients with rheumatoid arthritis
Source: Arthritis Res Ther. 2017 May 31;19:111. doi: 10.1186/s13075-017-1308-y (PMC5452609; doi:10.1186/s13075-017-1308-y)
Supplement: Supplementary file 1 — Comparison of age, sex, and BMI between rheumatoid arthritis (RA) patients and healthy donors (HDs). Data are indicated as mean +/- SD. Age and BMI were compared by the unpaired t test, and sex distribution was compared by chi-square test. Table S2. List of the primers (DOCX 15 kb) [file 13075_2017_1308_MOESM1_ESM.docx]

**Table S1**

|  | RA | HD | P |
| --- | --- | --- | --- |
| Age | 61.6 ± 12.8 | 38.8 ± 10.6 | <0.0001 |
| Female (%) | 75.3 | 79.8 | 0.59 |
| BMI | 23.1 ± 3.96 | 20.9 ± 3.07 | <0.0001 |

**Table S2**

| **Gene symbol** | **Forward primer** | **Reverse primer** |
| --- | --- | --- |
| CSF2 | [CATGTGAATGCCATCCAGGA](http://www.ncbi.nlm.nih.gov/entrez/query.fcgi?cmd=search&db=Nucleotide&dopt=GenBank&term=CAT%20GTG%20AAT%20GCC%20ATC%20CAG%20GA) | [CAGGCCCACATTCTCTCACTT](http://www.ncbi.nlm.nih.gov/entrez/query.fcgi?cmd=search&db=Nucleotide&dopt=GenBank&term=CAG%20GCC%20CAC%20ATT%20CTC%20TCA%20CTT) |
| RORC | CGCTCCAACATCTTCTCC | CTAACCAGCACCACTTCC |
| IL-17A | CAACCGATCCACCTCACCTT | GGCACTTTGCCTCCCAGAT |
| IL-17F | TGCCAGGAGGTAGTATGAAGCTT | ATGCAGCCCAAGTTCCTACACT |
| IL-22 | GCTTGACAAGTCCAACTTCCA | GCTCACTCATACTGACTCCGTG |
| CCL3 | GCAACCAGTTCTCTGCATCA | TGGCTGCTCGTCTCAAAGTA |
| CCL4 | GCTTTTCTTACACTGCGAGGA | CCAGGATTCACTGGGATCAG |
| CCL5 | CTACTCGGGAGGCTAAGGCAGGAA | GAGGGGTTGAGACGGCGGAAGC |
| AHR | AACAGATGAGGAAGGAACAGAGC | GAGTGGATGTGGTAGCAGAGTC |
| IL-10 | GAGATGCCTTCAGCAGAGTGAAGA | AGGCTTGGCAACCCAGGTAAC |
| MAF | CAAGCTAGAAGCGCCCC | AGTTCTGATGCCATTCTCCTG |
| GAPDH | GCTCTCCAGAACATCATCCCTGCC | CGTTGTCATACCAGGAAATGAGCTT |
