# Supplementary figures and images for: Increased serum concentrations of IL-1 beta, IL-21 and Th17 cells in overweight patients with rheumatoid arthritis
Source: Arthritis Res Ther. 2017 May 31;19:111. doi: 10.1186/s13075-017-1308-y (PMC5452609; doi:10.1186/s13075-017-1308-y)

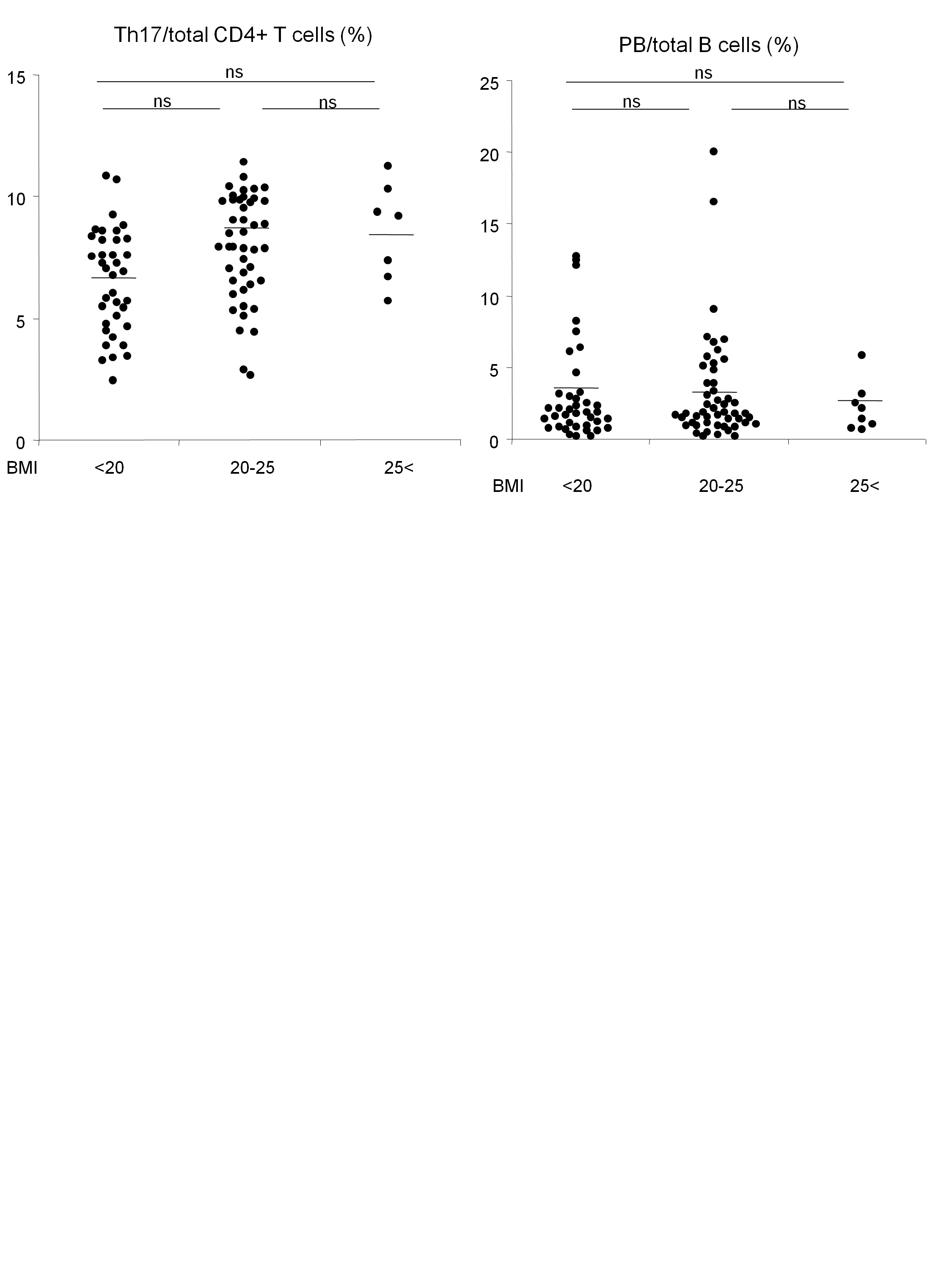

Supplement: Supplementary file 2 — Frequencies of peripheral immune cells (Th17 cells and Plasmablast (PB)) and BMI. A comparison of the frequencies of Th17 cells and PB between the three BMI groups among healthy donors. A p value <0.05 was defined as a significant difference. ns not significant (TIF 569 kb) [file 13075_2017_1308_MOESM2_ESM.tif]
